# Respiratory influence on cerebrospinal fluid flow – a computational study based on long-term intracranial pressure measurements

**DOI:** 10.1038/s41598-019-46055-5

**Published:** 2019-07-05

**Authors:** Vegard Vinje, Geir Ringstad, Erika Kristina Lindstrøm, Lars Magnus Valnes, Marie E. Rognes, Per Kristian Eide, Kent-Andre Mardal

**Affiliations:** 1 Department of Scientific Computing and Numerical Analysis, Simula Research Laboratory, 1325 Lysaker, Norway; 2 Institute of Clinical Medicine, Faculty of Medicine, University of Oslo, 0315 Oslo, Norway; 3 Department of Neurosurgery, Oslo University Hospital - Rikshospitalet, 0372 Oslo, Norway; 4 Department of Mathematics, University of Oslo, 0315 Oslo, Norway; 5 Department of Radiology and Nuclear Medicine, Oslo University Hospital - Rikshospitalet, 0372 Oslo, Norway

**Keywords:** Biophysical models, Computational models, Biophysical models

## Abstract

Current theories suggest that waste solutes are cleared from the brain via cerebrospinal fluid (CSF) flow, driven by pressure pulsations of possibly both cardiac and respiratory origin. In this study, we explored the importance of respiratory versus cardiac pressure gradients for CSF flow within one of the main conduits of the brain, the cerebral aqueduct. We obtained overnight intracranial pressure measurements from two different locations in 10 idiopathic normal pressure hydrocephalus (iNPH) patients. The resulting pressure gradients were analyzed with respect to cardiac and respiratory frequencies and amplitudes (182,000 cardiac and 48,000 respiratory cycles). Pressure gradients were used to compute CSF flow in simplified and patient-specific models of the aqueduct. The average ratio between cardiac over respiratory flow volume was 0.21 ± 0.09, even though the corresponding ratio between the pressure gradient amplitudes was 2.85 ± 1.06. The cardiac cycle was 0.25 ± 0.04 times the length of the respiratory cycle, allowing the respiratory pressure gradient to build considerable momentum despite its small magnitude. No significant differences in pressure gradient pulsations were found in the sleeping versus awake state. Pressure gradients underlying CSF flow in the cerebral aqueduct are dominated by cardiac pulsations, but induce CSF flow volumes dominated by respiration.

## Introduction

The interplay between intracranial pressure (ICP) and cerebrospinal fluid (CSF) flow plays an important role in e.g. cerebral homeostasis^1^, neurological conditions such as idiopathic normal pressure hydrocephalus (iNPH)^2^, and cerebral metabolic waste clearance^3^. For instance, in the context of iNPH patients, ICP amplitudes, CSF flow quantities such as aqueductal stroke volumes (ASVs), and auxiliary quantities such as compliance, have all been considered for predicting clinical response to shunt surgery^4^. According to the traditional view (the *third circulation*) introduced by Cushing in 1925^5^, CSF is mainly produced in the choroid plexus of the four ventricles and is mainly absorbed into the venous system through the arachnoid granulations. Over the last century and to a great extent in the recent decades, the classical view has been challenged–in particular in terms of CSF production^6^, routes and modes of (re-)absorption^3,7^, the drivers of CSF pulsatility^4^, and the effect of sleep^8,9^.

Over the last 30 years, cranial CSF flow has been studied extensively and non-invasively via PC-MRI techniques^10–15^. Using cardiac-gated MRI, it was early shown that cardiac-induced CSF flow pulsations vastly dominate the flow involved in the third circulation^10^. Later, respiration was also identified to influence CSF flow^16^. However, the relative importance of the cardiac and respiratory cycle is debated. Recent experimental studies have resulted in disparate findings: comparable contribution of respiration and cardiac pulsations to CSF velocities^13^, a significantly greater cardiac CSF velocity component^17^, and, conversely, inspiration as the most important driving force for CSF flow^12^. On the subject of a potential net CSF flow (in contrast or rather in addition to the pulsatile behavior), the current understanding is also incomplete. Cardiac-gated PC-MRI investigations have indicated considerable net CSF flow (vastly larger than the 0.5 L/day supposedly involved in the classical third circulation view), and moreover shown considerable variations between iNPH patients and controls^14,18^. On the other hand, there is evidence that net CSF flow in the cerebral aqueduct is confounded by the respiratory cycle^15,19^, questioning the validity of net CSF flow measured with cardiac gated PC-MRI.

In contrast, it is well-established that the dominating component of the ICP is the pressure pulsation of the cardiac cycle while the respiratory pulsation is considerably smaller^20^. In iNPH patients, the mean ICP wave amplitude related to the cardiac cycle has proved useful in predicting responders from surgery^21^. Static transmantle pressure gradients have not been demonstrated^22,23^. However, the classical view of the third circulation as well as recent findings of net CSF flow within the cerebral aqueduct^14^ suggest the existence of a static transmantle pressure gradient in addition to a pulsatile component. The existence of pulsatile pressure gradients has been hypothesized–as indeed suggested by the cardiac and respiratory CSF flow cycles. ICP differences in the cerebral aqueduct have been estimated from PC-MRI flow measurements^24,25^. However, very few have studied pulsatile ICP gradients clinically and directly^22^. Furthermore, the respiratory component in the ICP (gradient) signal has so far received very limited attention.

As the conflicting evidence shows, the current understanding of cardiac and respiratory influences on CSF flow and ICP, and on the relationship between the ICP and PC-MRI modalities, is inadequate. However, from a biomechanical point of view, the relationship between CSF flow and ICP gradients is governed by the Navier–Stokes equations and the CSF flow induced by pulsating (cardiac and/or respiratory) or near steady (third circulation) pressure gradients can readily be computed. Indeed, from computational studies it is well-known that the magnitude of CSF pressure gradients is only a small fraction of the ICP pulsation (typically >5 mmHg during a cardiac cycle)^2^. For example, a 42 *μ*L aqueductal stroke volume was estimated to correspond to an approximately 0.01 mmHg pressure drop in both a rigid and a deformable normally shaped patient-specific geometry^26^, while other computational studies have estimated a transmantle pressure difference of up to 0.03 mmHg^24,27^. Severely stenosed aqueducts may however have pressure drops that are orders of magnitude higher^28^.

**Figure 1:**
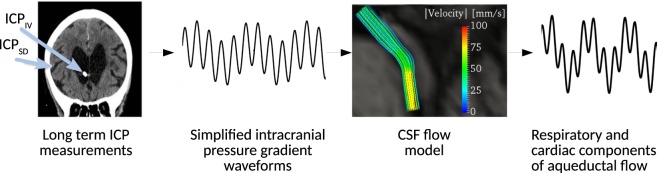
A schematic description of the steps involved in the present study. Subdural and ventricular long term ICP recordings were obtained *in vivo* and subtracted to find the ICP gradient. To compute aqueductal flow in both simplified and patient-specific geometries, a simplified ICP gradient consisting of two frequencies were constructed: one for the cardiac, and one for the respiratory component. The resulting flow rate was analyzed to compute peak volumetric flow rates as well as aqueductal stroke- and respiratory volumes.

On this background, the aim of this study was to investigate the relative importance of cardiac and respiratory contributions to ICP gradients, CSF flow rates and CSF flow volumes, and their interplay. An overview of the steps involved in the present study is shown in Fig. 1. In particular, we aimed to characterize the CSF flow induced by pulsatile cardiac and respiratory ICP gradients. We used a unique set of long-term *in-vivo* ICP measurements from two different intracranial locations in a cohort of 10 iNPH patients, to compute intracranial pressure gradients (dICPs). The ICP recordings were obtained from overnight registrations of patients breathing freely, and by extracting multiple 6-minute windows, a typical acquisition time of a PC-MRI scan. The dICP was subsequently used as the driving force in fluid dynamics models of the cerebral aqueduct. To utilize all the input data from the dICP recordings, an analytical solution of the flow field in a simplified geometry was first used and finally some of the input data was used on patient-specific geometries as well. In both models, we measured the cardiac- and respiratory induced peak volumetric flow rate (PVF), as well as the aqueductal stroke volume (ASV) and the corresponding aqueductal respiratory volume (ARV). The dICP data of each patient was separated into different time-frames to assess the effect of sleep on dICP amplitude and frequency. Finally, we compared the pressure gradients involved in the cardiac and respiratory cycles with the static pressure gradients involved in the third circulation.

## Results

### Quantification of pulsatile intracranial pressure gradients

**Figure 2:**
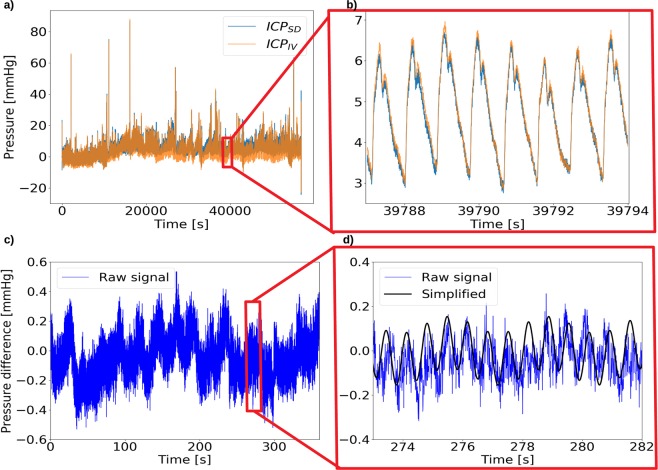
(**a**) Raw subdural ICP (ICP_SD_ (blue)) and intraventricular ICP (ICP_IV_ (orange)) for patient 1 (PatID 1) over the entire measuring period, before window selection. (**b**) A zoom of (**a**), demonstrating the small difference between the subdural and intraventricular ICP relative to ICP amplitudes (the latter typically being 4–5 mmHg). (**c**) An accepted 6-minute window for patient 1 after a shift to zero mean pressure difference. dICP amplitudes are considerably smaller than the ICP amplitudes. (**d**), a zoom of (**c**), showing the pressure difference in blue (difference between the two raw signals) and the simplified pressure difference (black) $\frac{\partial p}{\partial z}L$ derived from Eq. (2).

We analyzed the ICP measurements, as shown for patient 1 in Fig. 2a,b (and for all patients in Supplementary Fig. S1), by computing pulsatile ICP gradients (dICPs) as the difference between the two pressure sensor measurements divided by the distance between the sensors, and extracted sets of 6-minute windows of the resulting dICP waveforms (Fig. 2c). A total of 502 accepted 6-minute windows, consisting of approximately 182,000 cardiac cycles and 48,000 respiratory cycles were retrieved from the patients. For one patient (patient 10), the pressure window extraction resulted in only one accepted time window. This individual was excluded, leaving 9 patients for further study.

**Figure 3:**
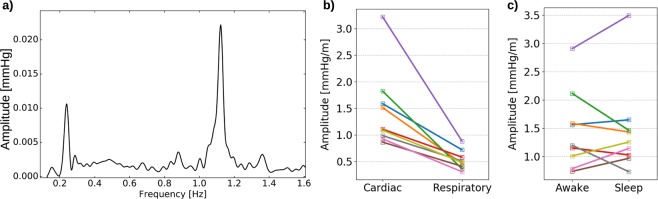
(**a**) The power spectrum of the pressure difference in an accepted 6-minute window from patient 1 after noise removal. Two peaks are evident, one related to the cardiac cycle (~1.1 Hz) and one related to the respiratory cycle (~0.2 Hz). Low frequencies (<0.1 Hz) are not shown. (**b**) Average dICP amplitude for each patient, cardiac vs respiratory. All patients had lower respiratory amplitudes on average. (**c**) Average cardiac dICP amplitude for each patient during the awake and sleeping state. Although some variations were found from patient to patient, no clear trend was evident.

We subsequently computed the power spectra of the 6-minute window dICP waveforms to identify and quantify the dominant signal frequencies and amplitudes (Fig. 3, Table 1). The waveforms consistently displayed two main frequency peaks, one related to the cardiac cycle and one to the respiratory cycle (Fig. 3a). The average heart rate ranged from 50 to 78 beats/minute for the 9 patients, while the respiratory period ranged from 13 to 17 cycles/minute (Table 1). At the cohort level, the (average ± standard deviation) heart rate was 62 ± 9 per minute and the respiratory rate was 15 ± 1 per minute. The average cardiac and respiratory periods plus/minus one standard deviation stayed well within the selected range defining the respiratory and heart rates (cf. Methods).

**Table 1:** Statistics from the dICP analysis.

PatID	N	L (cm)	Heart rate (Beats per minute)	Respiratory rate (Breaths per minute)	Cardiac pressure gradient, *a*_1_ (mmHg/m)	Respiratory pressure gradient, *a*_0_ (mmHg/m)	Pressure gradient ratio *a*_1/_*a*_0_
1	21	6.8	78 ± 9	17 ± 2	1.59 ± 0.47	0.72 ± 0.23	2.22
2	56	5.5	51 ± 9	15 ± 4	1.52 ± 0.85	0.46 ± 0.12	3.31
3	53	7.0	67 ± 2	15 ± 3	1.83 ± 0.74	0.36 ± 0.09	5.11
4	105	5.3	59 ± 4	13 ± 3	1.11 ± 0.47	0.58 ± 0.27	1.91
5	89	3.2	59 ± 4	14 ± 3	3.23 ± 1.12	0.88 ± 0.29	3.67
6	55	7.0	74 ± 7	17 ± 3	0.86 ± 0.23	0.41 ± 0.13	2.13
7	60	7.9	53 ± 7	17 ± 4	0.93 ± 0.52	0.30 ± 0.12	3.06
8	27	5.7	54 ± 3	15 ± 3	0.99 ± 0.61	0.51 ± 0.23	1.94
9	36	5.3	64 ± 4	16 ± 4	1.09 ± 0.43	0.48 ± 0.17	2.26
**Avg**.	**55**	**6**.**0**	**62** ± **9**	**15** ± **1**	**1.46** ± **0.74**	**0.52** ± **0.18**	**2**.**85** ± **1**.**06**

The last row shows average and standard deviation of average values from each individual patient. All values in the table have been rounded after as exact computations as possible. N: number of accepted 6-minute windows. L: distance between ICP sensors.

The signals persistently displayed pulsatile ICP gradients with both cardiac and respiratory contributions (Figs 2d and 3a). The average dICP amplitudes showed variability between patients, ranging from 0.86 to 3.23 mmHg/m for the cardiac component, and from 0.30 to 0.88 mmHg/m for the respiratory cycle (Fig. 3b, Table 1). The average cardiac dICP amplitude dominated the respiratory dICP amplitude by a factor of 2.85, with patients having average factors between 1.91 and 5.11 and a cohort standard deviation of 1.06.

### Comparing intracranial pressure gradients between sleep and wakefulness

Sleep has been reported to affect multiple aspects of cerebral fluid dynamics including solute transport^8^ and blood flow^9^. We therefore analyzed the intracranial pressure gradients further to investigate whether the cardiac and respiratory contributions differ between the sleep and awake state. To this end, we categorized all 6-minute windows as belonging to either the sleeping or awake state, and computed the average cardiac and respiratory amplitude and frequency for each state in each patient. No statistical differences were found in the sleeping versus awake state in any of these parameters (Fig. 3c, paired t-test, p > 0.5 for all parameters).

### Cardiac-dominated ICP gradients induce evenly distributed flow rates and peak velocities

In our data (Table 1), the cardiac contribution to the pulsatile ICP gradient dominates the respiratory component. On the other hand, other studies have reported that respiration is a substantial regulator of CSF flow^12,13^. Aiming to reconcile these observations via computational modelling, we investigated the fluid flow that would be induced by pulsatile intracranial pressure gradients. In particular, we assessed the CSF flow that could be induced in a cerebral aqueduct as a result of cardiac and respiratory ICP gradients.

We first considered simplified, two-frequency dICP waveforms by combining the cardiac and respiratory components of the pulsatile ICP gradients–for all 6-minute time windows in all patients. Using the simplified waveform as a driving force, we estimated the CSF velocities in a cylindrical model of the cerebral aqueduct using the incompressible Navier–Stokes equations. We subsequently computed the cardiac- and respiratory-induced volumetric flow rates over time and cardiac and respiratory peak volumetric flow rates (PVFs).

**Figure 4:**
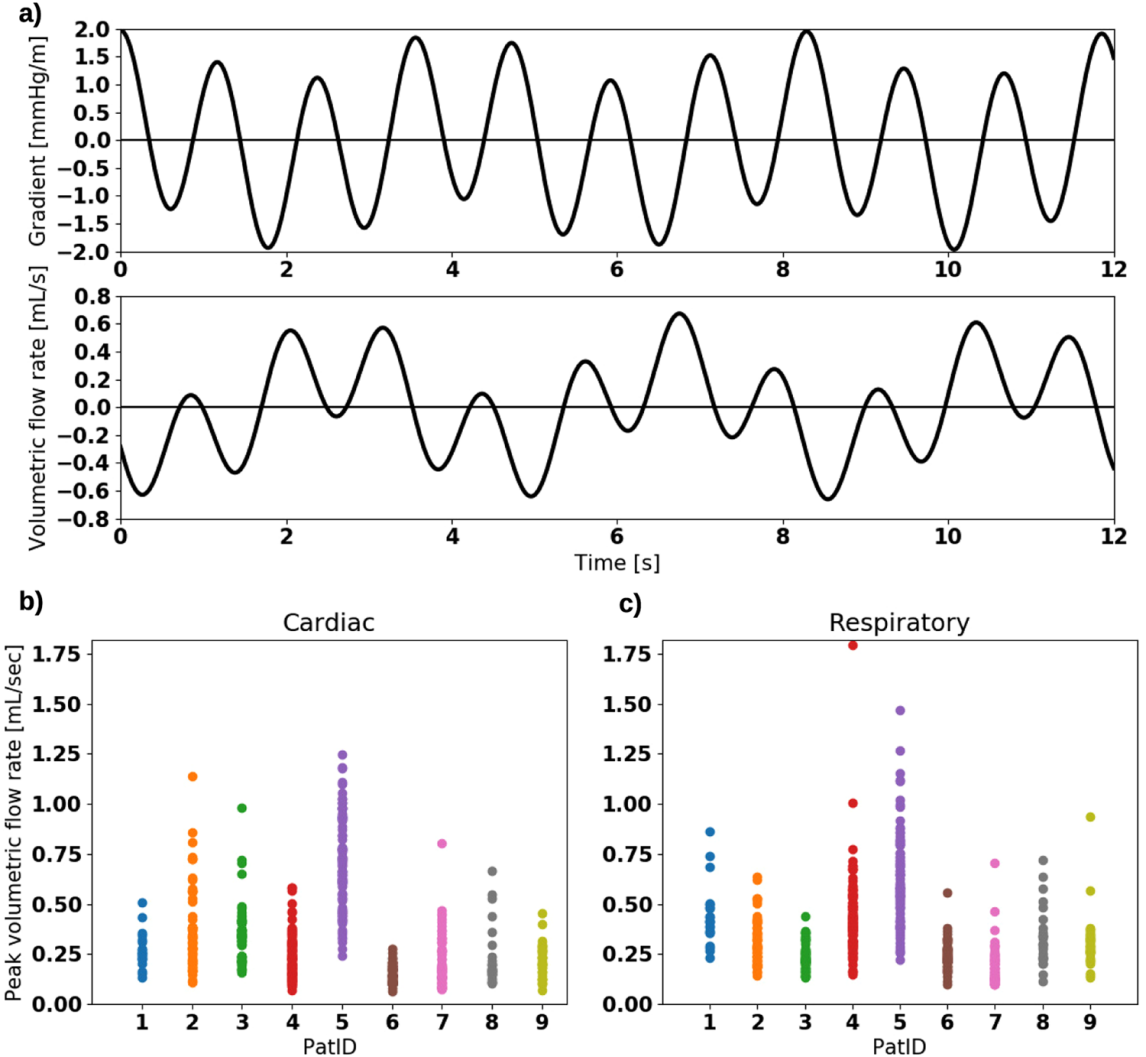
(**a**) Curves demonstrating average simplified pressure gradient and the computed volumetric flow rate through the cerebral aqueduct versus time for patient 2. The respiratory component is more prominent in the flow rate curve. For this patient, the ratio between cardiac and respiratory amplitude was 3.31 for the pressure gradient and 1.27 for the volumetric flow rate. (**b**) Cardiac peak volumetric flow rate for all patients over all time windows. (**c**) Respiratory peak volumetric flow rate for all patients over all time windows.

Figure 4a shows the simplified dICP waveform and the induced volumetric flow rate for the average cardiac and respiratory ICP gradients for patient 2. In this example, the dICP waveform is dominated by the contribution from the cardiac cycle: the cardiac dICP amplitude was 3.31 times the respiratory dICP amplitude (Table 1, patient 2). However, the volumetric flow rate induced by the dICP waveform was close to evenly regulated by the two frequencies. The ratio between cardiac and respiratory PVF was 1.18, with a cardiac component of 0.38 mL/s and a respiratory component of 0.32 mL/s (Table 2, patient 2).

**Table 2:** Statistics from the flow computations in the simplified cerebral aqueduct.

PatID	Cardiac PVF (mL/s)	Respiratory PVF (mL/s)	ASV (*μ*L)	ARV (*μ*L)	PVF ratio (*A*_1_/*A*_0_)	Ratio ASV/ARV
1	0.27 ± 0.09	0.44 ± 0.16	68.1 ± 27.8	523.3 ± 219.5	0.61	0.13
2	0.38 ± 0.21	0.32 ± 0.11	144.4 ± 80.8	464.2 ± 250.4	1.18	0.31
3	0.35 ± 0.15	0.24 ± 0.07	101.2 ± 46.7	330.1 ± 134.5	1.46	0.31
4	0.24 ± 0.11	0.43 ± 0.20	78.9 ± 38.7	695.6 ± 331.0	0.55	0.11
5	0.70 ± 0.23	0.62 ± 0.24	228.0 ± 79.2	929.6 ± 438.7	1.11	0.25
6	0.15 ± 0.04	0.25 ± 0.07	40.2 ± 13.7	303.7 ± 124.2	0.61	0.13
7	0.22 ± 0.13	0.19 ± 0.10	83.9 ± 52.5	255.2 ± 188.1	1.16	0.33
8	0.23 ± 0.15	0.33 ± 0.15	83.2 ± 55.9	433.8 ± 203.6	0.70	0.19
9	0.22 ± 0.08	0.31 ± 0.13	65.6 ± 25.0	404.9 ± 262.6	0.71	0.16
**Avg**.	**0.31 **±** 0.16**	**0.35 **±** 0.13**	**99.3 **±** 56.0**	**482.3 **±** 212.9**	**0**.**90** ± **0**.**33**	**0**.**21** ± **0**.**09**

The last row shows average and standard deviation of average values from each individual patient. All values in the table have been rounded after as exact computations as possible. PVF: peak volumetric flow rate. ASV: aqueductal stroke volume. ARV: aqueductal respiratory volume.

In the cohort in general, the cardiac-dominated ICP gradients induced cardiac and respiratory PVFs of comparable magnitudes (Table 2). The cohort had a cardiac PVF of 0.31 ± 0.16 mL/s and a respiratory PVF of 0.35 ± 0.13 mL/s. The individual average PVFs varied between 0.15 and 0.70 mL/s for the cardiac, and 0.19 and 0.62 mL/s for the respiratory cycle (Fig. 4c). The cohort ratio between cardiac and respiratory PVF was 0.90 ± 0.33, ranging individually from 0.55 to 1.46. We also observed that the peak velocity (in any location of the cerebral aqueduct and at any time point) typically reached 5–6 cm/s, with equal contribution from the cardiac and respiratory cycle. The cross-sectional average of the velocity reached only half of this value, approximately 3 cm/s.

### Evenly distributed CSF flow rates induce respiratory-dominated aqueductal flow volumes

Aqueductal stroke volume (ASV) has been proposed as a non-invasive marker of shunt response in iNPH. However, a challenge for the use of ASV derived from cardiac-gated PC-MRI is the effect of respiration, as respiration is traditionally not controlled for. To evaluate the effect of respiration on aqueductal flow volumes, we also computed the cardiac aqueductal flow volume, corresponding to the aqueductal stroke volume (ASV), and respiratory aqueductal flow volume (ARV) in the simplified model for all 6-minute time windows in all patients.

Interestingly, the computed flow volumes demonstrated a clear dominance of the respiratory component (Table 2). At the cohort level, the computed ASV was 99.3 ± 56.0 *μ*L. The average ASV in each patient ranged from 40.2 to 228.0 *μ*L. In comparison, the cohort-average ARV was 482.3 ± 212.9 *μ*L and individual patient average values ranged from 255.2 to 929.6 *μ*L. The ratio between ASV and ARV was 0.21 ± 0.09 and ranged from 0.11 to 0.33 between patients.

In addition to inter-individual variations, there was also variability in the computed flow volumes throughout the recording time within individuals (Table 2). At the patient level, the average standard deviation of ASV between different 6-minute windows was 46.7 *μ*L and ranged from 13.7 to 80.8 *μ*L, while the average ARV standard deviation was 239.2 *μ*L and ranged from 124.2 to 438.7 *μ*L. Thus, the variability in aqueductal flow volumes within a patient and between patients was comparable: the ratio between the average standard deviation within a patient to the standard deviation between patients was 0.83 for the ASV and 1.12 for the ARV.

### Evaluation of pulsatile ICP gradient induced flow for patient-specific geometries

To evaluate our findings for more complicated geometries, we next considered representations of the cerebral aqueduct of three patients diagnosed with iNPH. For each of the three patient-specific 3D geometries, we first simulated the CSF flow and pressure using aqueductal flow rates obtained from PC-MRI as boundary conditions for the incompressible Navier–Stokes equations, and computed the wave characteristics (amplitude and frequency) of the resulting simulated (cardiac) pressure gradients. Next, we added a custom respiratory pressure gradient to each of these simulated cardiac pressure gradients (cf. Methods and below). Finally, we simulated the total (cardiac and respiratory) pressure driven CSF flow in the three patient-specific aqueducts, and computed cardiac and respiratory PVFs, ASVs and ARVs.

Table 3 lists data obtained from PC-MRI for the three patients. The ASVs were measured to be 30.9, 94.7 and 110.4 *μ*L, and the heart rates were 103, 77 and 74 beats per minute. The first simulations, using flow obtained with PC-MRI as input data, gave (cardiac) pressure gradients with pulse amplitudes of 1.65, 4.51 and 2.92 mmHg/m, respectively. Based on the average cardiac-to-respiratory pressure gradient amplitude and frequency ratios (Table 1), respiratory amplitudes were assumed to be 2.85 times smaller, and rates 4.11 times lower. The respiratory pressure gradient amplitudes were thus estimated to be 0.58, 1.58 and 1.02 mmHg/m with rates of 25, 19 and 18 breaths per minute in the three patients.

**Table 3:** Heart rate, cardiac PVF and ASV obtained with data from the previous PC-MRI study.

PatID	Heart rate (beats per minute)	Respiratory rate ^#^(breaths per minute)	Cardiac PVF (mL/s)	ASV (*μ*L)	Cardiac gradient*(mmHg/m)	Respiratory gradient^#^ (mmHg/m)	Static gradient* (mmHg/m)
11	103	25	0.16	30.9	1.65	0.58	0.006
13	77	19	0.35	94.7	4.51	1.58	0.015
21	74	18	0.39	110.4	2.92	1.02	0.005
**Avg**.	**85 ± 16**	**21 ± 4**	**0**.**30 ± 0**.**13**	**78**.**7 ± 42**.**1**	**3**.**03 ± 1**.**43**	**1**.**06 ± 0**.**50**	**0**.**009 ± 0**.**006**

The cardiac induced pressure gradient was computed with computational fluid dynamics. The respiratory rate and gradient were estimated on the assumption that the three patients in the PC-MRI study had the same ratio between the cardiac and respiratory components as the 9 patients included in the dICP analysis. The last row shows average and standard deviation of average values from each individual patient. All values in the table have been rounded after as exact computations as possible. PVF: peak volumetric flow rate. ASV: aqueductal stroke volume. *Estimated from computational fluid dynamics. ^#^Estimated from average ratios between cardiac and respiratory frequency or gradients from ICP recordings.

**Figure 5:**
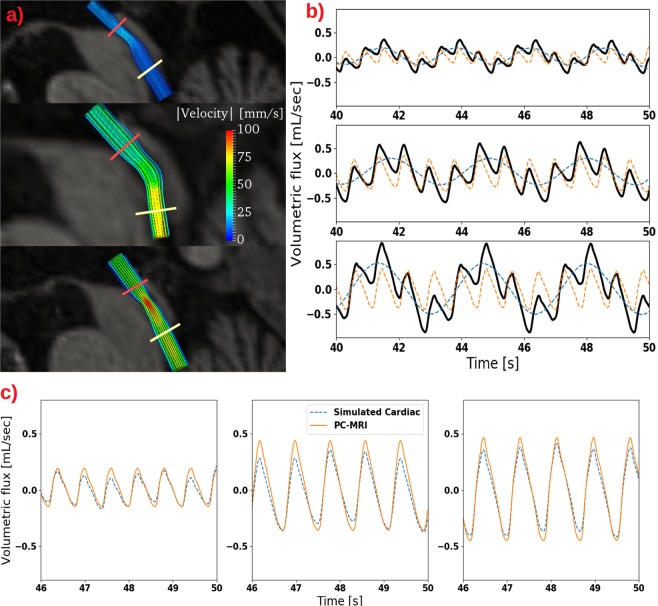
(**a**) Velocity distribution in the patient-specific 3D geometries at the time of peak velocity in all three patients (PatID11, PatID13, PatID21 from top to bottom). Peak velocity, flow rate, and spatial distribution of the flow field differed between the three geometries. Pressure gradients listed in Table 3 were computed in the physical part of the aqueduct, i.e. as a linear approximation between the two slices depicted in red and yellow. (**b**) Volumetric flow rate (black curve) as a function of time also decomposed into its cardiac (orange) and respiratory (blue) component. A prominent respiratory component of the flow pattern is present. (**c**) Comparison of flow rates obtained with PC-MRI with the cardiac component of the flow rate computed from pressure driven flow in the patient-specific geometries. The good agreement is as expected, however in contrast to the simplified model, respiratory pressure gradients may slightly affect cardiac flow due to nonlinearities in Navier-Stokes equations.

The sum of these pressure gradients, applied as pressure boundary conditions, induced laminar flow in all three patient-specific geometries, but differing in magnitude and distribution (Fig. 5). The simulated cardiac and respiratory PVF were similar to the flow rates in the simplified model (0.31 ± 0.16 vs 0.29 ± 0.13 mL/s for the cardiac, and 0.32 ± 0.17 vs 0.35 ± 0.13 mL/s for the respiratory PVF (Tables 2 and 4)). Moreover, the simulated ASVs were again dominated by the ARVs: the ASV was 69.9 ± 36.6 *μ*L, and ARV 308.1 ± 204.8 *μ*L. On average, the ratio between cardiac and respiratory components were 0.92 ± 0.23 for cardiac PVF versus respiratory PVF, and 0.24 ± 0.06 for ASV versus ARV (Table 4). We note that the final simulated cardiac PVFs and ASVs were slightly lower than the original (cardiac) PVF and ASV PC-MRI measurements (Tables 3 vs 4, also illustrated in Fig. 5c).

**Table 4:** Statistics from pulsatile flow computations in the patient-specific geometries.

PatID	Cardiac PVF (mL/s)	Respiratory PVF (mL/s)	ASV (*μ*L)	ARV (*μ*L)	PVF ratio (*A*_1_/*A*_0_)	Ratio ASV/ARV
11	0.14	0.18	28.8	127.1	0.81	0.23
13	0.32	0.27	82.4	266.8	1.17	0.31
21	0.39	0.51	98.6	530.4	0.76	0.19
**Avg**.	**0**.**29 ± 0**.**13**	**0**.**32 ± 0**.**17**	**69**.**9 ± 36**.**6**	**308**.**1 ± 204**.**8**	**0**.**92 ± 0**.**23**	**0**.**24 ± 0**.**06**

The last row shows average and standard deviation of average values from each individual geometry. All values in the table have been rounded after as exact computations as possible. PVF: peak volumetric flow rate. ASV: aqueductal stroke volume. ARV: aqueductal respiratory volume.

### Comparison of pulsatile and potential static ICP gradient magnitudes

When hydrostatic effects due to body position have been included, some authors have dismissed the idea of a transmantle pressure gradient^23^. If a net flow according to the third circulation exist, such a transmantle pressure gradient need to be present along the aqueduct. To investigate the magnitude of this potential static pressure gradient, we computed the pressure gradient needed to drive flow according to the third circulation.

The static pressure gradients that would result in a net flow of 500 mL/day through the cerebral aqueduct, as stipulated by the third circulation, were computed to be 0.009 ± 0.006 mmHg/m for the patient specific geometries (Table 3) and 0.0045 mmHg/m for the simplified geometry, with the latter computed by Poiseuille’s law. Thus, the measured pulsatile pressure gradients were approximately two orders of magnitude greater than these static pressure gradient estimates. We further remark that these estimates are five orders of magnitude lower than the pressure increase involved in infusion tests as described by Davson’s equation^29,30^.

## Discussion

The relative importance of cardiac and respiratory contributions to ICP and CSF fluid dynamics is not yet well understood. This computational study gives a mechanistic explanation for seemingly disparate experimental findings. In particular, we demonstrate that small cardiac-dominated pulsatile ICP gradients yield CSF peak flow rates with comparable cardiac and respiratory components, and respiratory-dominated aqueductal flow volumes. Compared to the cardiac period, the longer respiratory period allows momentum from the small respiratory pressure gradient to build up over time. These findings are consistent across both simplified and patient-specific geometries, and based on measurement data from more than 180,000 cardiac cycles and 48,000 respiratory cycles in 9 iNPH patients.

ICP has long been known to be pulsatile, and dominated by the cardiac component^31^. On the other hand, the existence of transmantle and/or intracranial pressure gradients has been much discussed. A transmantle pressure gradient has been proposed to be the mechanism behind enlarged ventricles in iNPH^22,32^. Stephensen *et al*.^23^ reported that no static transmantle pressure difference exists in iNPH, suggesting alterations in pulsatile rather than static pressure to explain ventricular enlargement. In a fluid-structure interaction model, Linninger *et al*.^33^ argued that pulsatile pressure gradients do not need to be large to produce ventricular enlargement. Our findings are consistent with these reports: our analysis revealed small pulsations also in the ICP difference between the ventricular CSF and subdural compartment, of both cardiac and respiratory origin, and dominated by the cardiac cycle.

The pulsatile ICP gradients were of comparable magnitude as previous analyses of pulsatile ICP measurements^22,34^. Except for one data point (Table 1, patient 5), pulsatile ICP gradients in our study were well within values (less than 3 mmHg/m) estimated non-invasively with PC-MRI^25,35^. The recorded pressure differences in our study were slightly higher than what was estimated in a computational model by Sweetman *et al*. (0.03 mmHg vs 0.1 mmHg in our study)^27^. The ICP gradients computed from the pressure difference signal were of similar magnitude as have been reported from simulations of CSF flow in the foramen magnum: Martin *et al*.^36^ used flow data to estimate a pulsatile gradient with a maximum value of 0.67 mmHg/m, while Rutkowska *et al*.^37^ used a pulsatile gradient of approximately 3.1 mmHg/m to compute pulsatile CSF flow. In our data, collected from a total of 502 6-minute windows in iNPH patients, ICP gradient variation over time was high compared to a recent MRI-study of healthy controls over four 6-minute windows^25^. The difference in cohort (iNPH patients vs healthy) and possibly difference in modality (long-term ICP monitoring vs MRI at the craniocervical region) and the total number of observations may explain this discrepancy. It is worth noting that the patient with the highest dICP also had the lowest distance between the two sensors. The distance between the two sensors affects the measured pressure difference, however no clear relationship between the distance between sensors and the computed pressure gradient was found.

In our study, we found the static pressure gradient needed to drive net CSF flow according to the traditional third circulation hypothesis^5^ (corresponding to a CSF production of 500 mL/day) to be extremely small (less than 0.01 mmHg/m), approximately two orders of magnitude lower than the measured pulsatile ICP gradients. Therefore, a transmantle pressure difference close to zero (of the order 0.01 mmHg) as found by Stephensen *et al*.^23^ is be sufficient to drive flow of comparable magnitude to CSF production. Accurate measurements of static ICP gradients of this size are hard to obtain in the clinical setting.

That respiration plays a significant role for the CSF flow compares well with recent studies, although the ratio between cardiac and respiration influence varies. Dreha-Kulaczewski *et al*.^12^ previously concluded that inspiration is the major regulator of CSF flow, and later proposed that inspiration-induced downward flow of venous blood is counterbalanced by an upward CSF movement with a respiratory volume of 300–600 *μ*L^38^. We find ARVs of comparable size, but in contrast to the latter study, we estimated flow during free breathing and not during a breathing protocol. For CSF velocities in the cerebral aqueduct, Takizawa *et al*.^17^ found the cardiac component to be approximately two times greater than the respiratory contribution. Similarly, Yildiz *et al*.^13^ found a factor two between the cardiac and respiratory components of the velocity in the foramen magnum.

MRI-studies have found CSF displacement to be dominated by respiration: the respiratory component has been found to be two to three^17^ and three to four^39^ times greater than the cardiac component. The displacement in these two studies is defined as the time integral of the measured velocity. Therefore, we would expect the ratio between velocity and displacement as measured in these two studies, to compare to the ratio between volumetric flux and volume as analyzed in our study. The computed ratio between ARV and ASV in our study aligns well with the aforementioned findings on CSF displacement.

Our findings on the variation and effect of respiration on aqueductal flow volumes are also interesting in a clinical perspective. The range of average ASVs in our study (in both simplified and patient-specific geometries) is in agreement with earlier findings^40–42^. We note that, in the simplified model, we found the average standard deviation within a patient over one day to be 39 *μ*L. This is a substantial variation, especially viewed in the context of the potential use of ASV as an indicator for selecting NPH patients for surgical shunting^40^. Intra-patient standard variation of 34 *μ*L in ASV has earlier been shown on the timescale of months^42^, but not on the short timescales provided in our study. A fundamental challenge to the use of ASV derived from cardiac gated PC-MRI may also be the effect of respiration, as respiration is traditionally not controlled for. Our findings show that selecting patients for shunt surgery based on the ASV alone may be inadequate if the pulsatile CSF volume through the cerebral aqueduct is the determining factor for shunt response. If this is the case, the ARV is a factor (at least) as important to consider as the ASV in selection of shunt responders.

ICP gradients of the (small) magnitude reported in this study are in general little studied, difficult to measure reliably, and subject to several potential sources of error. First, the raw data collected from the ICP sensors contained some high frequency noise of magnitude comparable to the physiological pulsation in the pressure gradient. Second, even though the sensors used in this study have been validated with respect to sampling rate^43^, the pressure differences measured (0.1–0.2 mmHg) are small compared to the amplitudes for which the validation took place (approximately 5 mmHg). Finally, we can not exclude the possibility that systematic differences between pairs of sensors (e.g. differences in sensor sensitivity) could introduce artificial oscillating pressure gradients of comparable magnitude to those reported here. That said, since new sensors are used each time, the systematic difference would only apply to individual investigations and not the group of patients as a whole. Further, these potential errors would directly affect the flow patterns. Clearly, an overestimation (resp. underestimation) of the actual ICP gradients would overestimate (resp. underestimate) flow rates and flow volumes, and likely shift the relative importance of cardiac and respiratory contributions. On the other hand, we argue that the following considerations add confidence to the reported ICP gradients. Our analysis compared 502 6-minute windows with approximately 182,000 cardiac cycles and 48,000 respiratory cycles in total. The large amount of observations gives confidence in the cardiac and respiratory pulsations observed in the pressure difference signal. In addition, we note that the measured pressure gradients are of the same frequency, and of similar amplitude to what has already been found or estimated in the literature in both PC-MR and simulation studies c.f. e.g.^25,27,28,35–37^. Finally, with regard to potential systematic sensor differences, we find such a difference unlikely to occur consistently in 9 patients over several hours for each patient.

The original data collection did not record body position, thus providing no information on possible hydrostatic transmantle pressure gradients. To remedy the lack of information, we applied a shift of the pressure difference, giving it a zero mean value, which has been reported to be the case when body position is taken into account^23^. This shift removes any constant-in-time pressure gradients, and in particular hydrostatic transmantle pressure gradients. Thus our simulations had no bulk flow, which otherwise would be superimposed on the pulsatile flow, not affecting the outputs measured or computed in this study. Similarly, in this study, we did not analyze other low frequency patterns (of frequency <0.1 Hz). Small head movements during measurements will cause sudden increase in the hydrostatic gradient between the two sensors, resulting in energy in the low-frequent region of the Fourier-spectrum. Low frequency components were removed using a fitted exponential function, therefore the respiratory magnitude may be slightly underestimated. As such, our respiratory amplitudes were conservatively estimated.

For the CSF flow simulations, we have assumed that the gradient in the cerebral aqueduct is equal to the measured pulsatile ICP gradient; i.e., the difference in the measured pressure divided by the distance between the sensors. We have thus assumed that the ICP varies linearly throughout the intracranial compartment and is equal in all directions. This stipulation clearly ignores existing heterogeneity in the parenchymal tissue and CSF compartments. On the other hand, pressure gradients in the two patients with sensors placed in the parenchyma instead of in the subdural space (PatID4 and PatID5) did not show any clear discrepancy between gradients from the rest of the cohort. In addition, we note that, under this assumption, the cardiac-induced pressure gradients found from the ICP measurements were similar to the pressure gradients found with CFD in the patient-specific 3D geometries using CSF flow measured with PC-MRI as input (1.46 vs 3.03 mmHg/m). To drive the same amount of flow, the pressure gradients in the narrow part of the aqueduct would be expected to be higher than in the straight tube representing the simplified aqueduct. In addition, we note that the heart rate was higher in the cohort that underwent PC-MRI, possibly affecting the cardiac induced pulsatile gradient amplitude as well. However, on cohort average, the ratio between cardiac and respiratory PVF as well as the ratio between ASV and ARV were almost identical in the simplified and in the patient-specific models

In terms of other limitations, in the simplified flow simulations, the cerebral aqueduct was assumed to be a rigid cylinder, equal in all patients. We note however, that the aqueduct radius differs between individuals, and is typically 1–3 mm in healthy^44^, while the median and range were approximately 2 mm, and 1.7–3.5 mm, respectively, in a cohort of 21 iNPH patients^41^. In earlier computer models, a radius below 1 mm has been used^28^. An increased radius would directly affect resistance and thus flow rate, ASV and ARV in our model. On the other hand, the relative importance of cardiac versus respiratory effects would be less affected as the Womersley number for both components will be linearly shifted with a change in radius. Possible effects of narrowing, curvature, or other geometrical differences were ignored in the simplified model. However, our findings were robust with regard to changes in geometry: a given ratio between cardiac and respiratory ICP gradients resulted in a similar ratio between cardiac and respiratory-induced flow rates and volumes in all (simplified and patient-specific) geometries. Also, the input pressure gradient was simplified as the sum of two sinusoidal waves representing the cardiac and respiratory cycle in the simplified model. Moreover, geometries and pressure recordings were obtained from iNPH patients only, where aqueductal flow may be hyperdynamic^40^.

In the patient-specific geometries, the time varying inlet boundary condition was assumed to be a function constant in space. However, the flow extensions ensured that flow had developed before reaching the narrow parts of the aqueduct. In the simulations, we also verified that the solutions were independent of the stabilization parameter, time-step and element size in the mesh: a 50% reduction in either of these parameters resulted in less than 2% change in peak volumetric flow rate.

For the categorization of sleeping versus awake states, we did not monitor whether the patients were actually sleeping at nighttime, but rather assumed a sleeping state at night hours. Thus, our quantification of differences between sleeping and awake states likely combined the effects of sleep and the sleep-independent circadian rhythm.

In conclusion, we have demonstrated, via fundamental mechanics and computational modelling, how a relatively small respiratory-induced ICP gradient pulsation induced flow volumes that dominated the cardiac component in the cerebral aqueduct. Our study supports the notion that respiration contributes substantially to CSF flow, and suggests that respiration should be investigated as a potential driver of other forms of intracranial fluid flow such as e.g. paravascular flow or potential glymphatic circulation^45,46^.

## Methods

### Approvals

The simultaneous ICP measurements were performed in a study approved by The Regional Committee for Medical and Health Research Ethics (REK) of Health Region South-East, Norway (approval no. S-08670b) and the Institutional Review Board of Oslo University Hospital–Rikshospitalet (no. 08/6827). Patients were included after written and oral informed consent.

For acquisitions of PC-MRI and patient specific geometries (T1-weighted volume scans), approval was retrieved by the Regional Committee for Medical and Health Research Ethics (REK) of Health Region South-East, Norway (2015/96) and the Institutional Review Board of Oslo University Hospital (2015/1868) and the National Medicines Agency (15/04932-7).

All methods were performed in accordance with the relevant guidelines and regulations.

### Intracranial pressure monitoring and acquisition

In the Department of Neurosurgery at Oslo University Hospital–Rikshospitalet, overnight ICP monitoring is part of a standardized pre-operative protocol for iNPH patients. The results of ICP monitoring are among the criteria deciding which patients that are offered shunt surgery^2^. Measurements from two ICP sensors were part of a research protocol. In 10 iNPH patients, simultaneous ICP measurements were obtained overnight from two ICP sensors (Codman ICP microsensor, Raynham, MA, USA) within the intracranial compartment. In all patients, one sensor was placed within the lateral ventricle (ICP_IV_). In eight patients, the other sensor was placed in the subdural compartment (ICP_SD_) while in two patients (PatID4 and PatID5) it was placed in the parenchyma (ICP_PAR_). For further information on sensor placement, see the previous description^22^. The pressure sensors were placed in local anesthesia with the patient in the operating room. Following a small straight incision in the right frontal region of the head, a burr hole about 1 to 1.5 cm was made. Thereafter, a minor incision was made in the dura. An external ventricular drain (EVD) with a built-in Codman ICP MicroSensor (Codman external drainage with ICP sensor; Codman/Johnson & Johnson, Raynham, MA, USA) was placed within the frontal horn of the cerebral ventricles. The distal end of the EVD was placed at the level of foramen Monroi. Through the same burr hole, another Codman ICP MicroSensor (Codman, Johnson & Johnson, Raynham, MA, USA) was also placed between the arachnoidea and the dura. Both sensors were zeroed before implantation. The burr hole opening was closed with bone wax in order to avoid CSF leakage. When the patient had returned to the neurosurgical ward, the Codman ICP sensors were connected to Codman ICP Express (Codman/Johnson & Johnson, Raynham, MA, USA) and the continuous analogue ICP signals provided by Codman ICP Express were digitalized using the analogue-digital converter (Sensometrics Pressure Logger, Sensometrics software, dPCom, Oslo, Norway). Both continuous digital ICP signals were sampled simultaneously with identical time reference at a sampling rate of 200 Hz, and stored as rawdata files, using Sensometrics software. The sampling rate is sufficient for accurate assessment of ICP waveforms^47^. All patients undergoing ICP monitoring were breathing freely and not on artificial respiration.

### MRI acquisition

The MRI study consisted of three iNPH patients (PatID11, PatID13, PatID21) and was obtained for study purposes during the pre-operative protocol. From the three patients, we obtained T1-weighted images (used for mesh-construction) and PC-MRI to obtain cardiac-gated CSF flow. Details on the T1-weighted image and the PC-MRI acquisitions, the segmentation and mesh generation based on the T1-weighted images, and the post-processing of the PC-MRI images to obtain the time-varying flux are given in the Supplementary Material. The PC-MRI post-processing has also previously been described in detail^14^.

### Differential intracranial pressure analysis

We computed the ICP gradient (dICP) waveform as a function over time by the difference between the two pressure signals divided by the distance L between the sensors: dICP = (ICP_SD_ − ICP_IV_)/L. In the two patients with sensor placement in the parenchyma, the dICP was defined as dICP = (ICP_PAR_ − ICP_IV_)/L. To assess time variability within each patient, we extracted sets of 6-minute windows of the dICP waveforms. Six minutes is the typical duration of a cardiac-gated PC-MRI scan. A 6-minute window was accepted if the maximal variability in the pressure difference was less than 2 mmHg over the 6-minute window. As body position was not recorded, hydrostatic pressure may contribute to static pressure differences between the two sensors. To compensate, the dICP waveform was shifted to have zero mean within each 6-minute window.

For each dICP waveform 6-minute window, we computed its power spectrum using the fast Fourier transform to identify and quantify the dominant frequencies and amplitudes. A low-pass filter with cutoff frequency 15 Hz was applied to the power spectrum of the dICP waveform. The cardiac amplitude was defined as the peak between 0.7 and 1.6 Hz (42–96 beats per minute), while the respiratory amplitude was defined as the peak between 0.15 and 0.4 Hz (9–24 breaths per minute). For each patient, we extracted cardiac and respiratory amplitudes and the corresponding frequencies for all 6-minute windows associated with the given patient.

The power spectrum of the 6-minute windows of the dICP waveform also revealed low frequency (less than 0.1 Hz) patterns. We modeled the low frequency patterns as a decreasing exponential function fitted to the power spectrum function and subtracted this function from the original power spectrum for all 6-minute windows.

### Categorization of sleeping versus awake state

All 6-minute time windows were categorized as belonging to either the sleeping or awake state based on the time of recording: all windows between midnight and 06:00 am as sleeping, and all others as awake.

### Pulsatile CSF flow in a cylindrical geometry

To model CSF flow induced by the dICP in a simplified model of the cerebral aqueduct, we solved the incompressible Navier-Stokes equations with the dICP as a driving force. We modelled a simplified cerebral aqueduct as a rigid cylinder with radius R = 2 mm, with radial size motivated by a median cerebral aqueduct area of 14 mm^2^ in iNPH patients^41^. Under these assumptions, the incompressible Navier-Stokes equations reduce to a one-dimensional differential equation^48^:

1 $$\rho \frac{\partial v}{\partial t}(r,t)-\frac{\mu }{r}\frac{\partial v}{\partial r}(r,t)-\mu \frac{{\partial }^{2}v}{\partial {r}^{2}}(r,t)=-\,\frac{{\rm{d}}p}{{\rm{d}}z}(t)\mathrm{.}$$

Here, *ν* is the radially-varying velocity in the z-direction along the cerebral aqueduct for 0 ≤ *r* ≤ *R*, and time 0 ≤ *t* ≤ *T* for some final simulation time *T*, $\frac{{\rm{d}}p}{{\rm{d}}z}$ is the pressure gradient along the cerebral aqueduct (Pa/m), and *ρ* and *μ* are CSF density and viscosity, respectively.

For each 6-minute time window, we expressed the simplified pressure gradient as

2 $$\frac{{\rm{d}}p}{{\rm{d}}z}(t)={a}_{0}\,\sin (2\pi t{f}_{0})+{a}_{1}\,\sin \,\mathrm{(2}\pi t{f}_{1}\mathrm{).}$$

with frequencies *f*_0_ and *f*_1_ and amplitudes *a*_0_ and *a*_1_. We identified *f*_0_ and *f*_1_ as the frequency of the cardiac and respiratory peak in the dICP frequency spectrum, respectively. It has previously been shown that ICP amplitudes extracted directly from peak values in the frequency domain would underestimate amplitudes in the time domain^47^. To compensate, we multiplied the amplitudes extracted from the power spectrum by a factor C to compute the amplitudes in Eq. (2). A factor *C* = 7 was chosen heuristically to obtain time domain amplitudes of comparable size as the original raw signal (see Fig. 2d). This scaling does not affect the ratio between the cardiac and respiratory component in the dICP signal. We labeled *f*_0_ and *a*_0_ as the cardiac frequency and cardiac dICP amplitude, and *f*_1_ and *a*_1_ as the respiratory frequency and respiratory dICP amplitude.

The solution to Eq. (1) with a sinusoidal pressure gradient of amplitude *a* and frequency *f* can be calculated analytically^49^. The analytical flow rate is given by

3 $$Q(t)=Re\{\pi {r}^{2}\frac{i\,a}{\rho \omega }[1-\frac{2}{{\rm{\Lambda }}}\frac{{J}_{1}({\rm{\Lambda }})}{{J}_{0}({\rm{\Lambda }})}]{e}^{i\omega t}\}$$

where *r* is the radius of the cylinder, *a* is the pressure gradient amplitude, *ω* = 2*πf* is the angular frequency of the pressure gradient, and Λ = *αi*^3/2^, where $\alpha =r{(\frac{\omega \rho }{\mu })}^{\mathrm{1/2}}$ is the Womersley number and $i=\sqrt{-\,1}$. *J*_0_ and *J*_1_ are Bessel Functions of the first kind with order zero and one, respectively. The corresponding peak volumetric flux (PVF), is thus given by the amplitude of the signal:

4 $$A=|\pi {r}^{2}\frac{i\,a}{\rho \omega }[1-\frac{2}{{\rm{\Lambda }}}\frac{{J}_{1}({\rm{\Lambda }})}{{J}_{0}({\rm{\Lambda }})}]|.$$

By the linearity of Eq. (1), the pressure gradient from Eq. (2) will result in a sum of two flow rate functions with frequencies *f*_0_ and *f*_1_, and corresponding PVFs, *A*_0_ and *A*_1_, each given by Eq. (4).

For all 6-minute time windows in all patients, we used Eq. (4), with cardiac frequency *f*_0_ and respiratory frequency *f*_1_ as separate inputs, to compute the cardiac (*A*_0_) and respiratory PVF (*A*_1_). We further defined the cardiac component of the aqueductal flow volume - the aqueductal stroke volume (ASV), and the aqueductal respiratory flow volume (ARV) as *V*_0_ and *V*_1_ respectively, where

5 $${V}_{i}={\int }_{0}^{\frac{1}{2{f}_{i}}}\,{A}_{i}\,\sin (2\pi t{f}_{i}){\rm{d}}t=\frac{{A}_{i}}{\pi {f}_{i}}.$$

for *i* = 0, 1.

### PC-MRI guided CSF flow in patient-specific 3D geometries

Three patient-specific geometries were constructed using the software VMTK (1.4.0)^50^ together with MR images of patients diagnosed with iNPH (see also Section on MRI acquisition above). The process is described in the Supplementary Material, and visualized in Supplementary Fig. S2. Flow extensions were added to each end of the aqueduct geometry to minimize the effect of the choice of spatial inlet velocity profile, such that the flow was developed when it reached the narrow part of the aqueduct. The flow extensions were constructed by computing the center-lines from the inlet to the outlet, and adding cylinder extensions in the direction of the center-lines.

The following steps were then carried out for each of the three patient-specific geometries. To first estimate patient-specific pulsatile pressure gradients, given the PC-MRI flow rate measurements, we computed an inlet velocity by dividing the volumetric flow rates obtained from the PC-MRI by the inlet area of the patient-specific geometry. The PC-MRI flow data also revealed the cardiac frequency *f*_0_ in each case. We subsequently solved the 3D incompressible Navier-Stokes equations (in Cartesian coordinates) over the geometry with this time-varying velocity prescribed at the inlet, no-slip conditions at the rigid outer walls, and a zero pressure (pseudo-traction) condition at the outlet. The system started at rest, and the end time was *T* = 50 s with a time step of Δ*t* = 0.01 s. The equations were solved simultaneously for the CSF velocity and pressure with linear finite elements with a stabilization term on the mass conservation equation, using the FEniCS finite element software^51^. Results from the last 10 seconds of the simulations were used for post-processing, allowing for at least one full respiratory period.

From the first set of simulations, pressure gradients in the patient specific geometries were computed in the physical aqueduct, excluding the flow extensions as shown in Fig. 5 (and Supplementary Fig. S2). The resulting pressure difference between the two slices defining the physical part of the aqueduct was divided by the length of the aqueduct (center-line) *L*, $d{p}_{0}(t)=\frac{1}{L}({p}_{{\rm{in}}}(t)-{p}_{{\rm{out}}}(t))$ to compute the cardiac induced pressure gradient. The amplitude of the cardiac pressure gradient was then computed as ${a}_{0}=\frac{1}{2}({\rm{\max }}(d{p}_{0}(t))-\,{\rm{\min }}(d{p}_{0}(t))$. Given the pulsatile cardiac-induced pressure gradient, we computed a representative sinusoidal respiratory component $d{p}_{1}(t)={a}_{1}\,\sin \,\mathrm{(2}\pi {f}_{1}t)$ with frequency *f*_1_ = *βf*_0_ and amplitude *a*_1_ = *αa*_0_. We let *β* = 1/4.11 and *α* = 1/2.85 based on cardiac versus respiratory analysis of the ICP measurement cohort (cf. Table 1).

To investigate whether a given ratio between cardiac and respiratory pressure gradients would result in a given ratio between cardiac and respiratory flow, regardless of small changes in the geometry, we next computed pressure driven flow in the patient-specific models. To simulate pressure driven flow, the pressure difference between the inlet and outlet of the geometry, including flow extensions, are needed. Therefore, in addition to the pressure gradient in the physical aqueduct, we also computed the pressure difference between the inlet and outlet of the full geometry in the first set of simulations. Next, we again assumed the corresponding respiratory pressure difference to be a sinusoidal curve with 2.85 times smaller amplitude and frequency 4.11 times lower than the cardiac pressure difference. Finally, in the second set of simulations we prescribed the sum of the cardiac and respiratory pressure difference between the inlet and outlet as boundary conditions.

For the patient-specific geometries and simulations, we obtained the patients’ heart rate, PVF and ASV from the relevant PC-MRI (Table 3). When pressure driven flow was simulated, the volumetric flow rate Q, as a function of time, was calculated by integrating the velocity over the cross-section of the cerebral aqueduct at the outlet. To separate the respiratory and cardiac components of the flow rate curve, we defined the following procedure. We first defined all peaks and valleys of the flow rate curve *Q*(*t*) for the last 10 seconds of the simulation. For each pair (Qp, Qv) of peak and valley, we defined points (Qp + Qv)/2, and defined the respiratory volumetric flow rate function *Q*_*r*_(*t*) as a continuous interpolant of these points (see e.g. blue curves in Fig. 5). The cardiac component was then defined as the difference between the volumetric flow rate and its respiratory component, *Q*_*c*_(*t*) = *Q*(*t*) − *Q*_*r*_(*t*). The cardiac and respiratory PVFs were then defined as (max(*Q*_*i*_(*t*))- min(*Q*_*i*_(*t*))/2 for i = c,r. The ASV and ARV were computed by solving

6 $$[ASV,ARV]=\frac{1}{{N}^{i}}{\int }_{{t}_{0}^{i}}^{{t}_{1}^{i}}\,\frac{|{Q}_{i}(t)|}{2}{\rm{d}}\,t\,{\rm{for}}\,{\rm{i}}={\rm{c}},{\rm{r}}$$

where ${t}_{0}^{c}$ is the time of the first cardiac peak during the last 10 seconds, ${t}_{i}^{c}$ is the time of the last cardiac peak, and *N*_*c*_ is the number of full cardiac cycles over the last 10 seconds. ${t}_{0}^{r}$, ${t}_{1}^{r}$ and *N*^*r*^ are defined analogously for the respiratory cycle. We used the trapezoidal rule for numerical integration in time to compute the ARV and ASV given by Eq. (6).

### Static pressure gradients

In all geometries, we also computed the static pressure gradient involved in the third circulation^5^; i.e., the pressure gradient required to drive a net CSF flow of 500 mL/day^52^ through the cerebral aqueduct. In the simplified model, the pressure gradient was calculated by Poiseuille’s law. In the patient-specific models, we computed the pressure gradient in the aqueduct by solving Stokes equation with a constant inlet velocity corresponding to a net flow of 500 mL/day. The static pressure gradient for each geometry was computed in the physical part of the aqueduct, not including flow extensions, as described in the previous subsection.

## Supplementary information

Supplementary Material

## Data Availability

The datasets analyzed in the current study are available from the corresponding author upon request.
