# Supplementary material for: Respiratory influence on cerebrospinal fluid flow – a computational study based on long-term intracranial pressure measurements
Source: Sci Rep. 2019 Jul 5;9:9732. doi: 10.1038/s41598-019-46055-5 (PMC6611841; doi:10.1038/s41598-019-46055-5)
Supplement: Supplementary file 1 — Supplementary Material [file 41598_2019_46055_MOESM1_ESM.docx]

**Respiratory influence on cerebrospinal fluid flow – A computational study based on long-term intracranial pressure measurements**

Vegard Vinje^1,*^, Geir Ringstad^2,5^, Erika Kristina Lindstrøm^4^, Lars Magnus Valnes^4^, Marie E. Rognes^1^,

Per Kristian Eide ^2,3^, and Kent-Andre Mardal^1,4^

^1^Department of Scientific Computing and Numercial Analysis, Simula Research Laboratory, 1325 Lysaker, Norway.

^2^Institute of Clinical Medicine, Faculty of Medicine, University of Oslo, 0315 Oslo, Norwa.y

^3^Department of Neurosurgery, Oslo University Hospital - Rikshospitalet, 0372 Oslo, Norway.

^4^Department of Mathematics, University of Oslo, 0315 Oslo, Norway.

^5^Department of Radiology and Nuclear Medicine, Oslo University Hospital - Rikshospitalet, 0372 Oslo, Norway.

^*^vegard@simula.no

**Results:**

Supplementary Figure S1 shows the difference between subdural and ventricular pressure in all 9 patients. Whether the transmantle pressure difference is negative or positive varies from patient to patient, and in some patients over time.


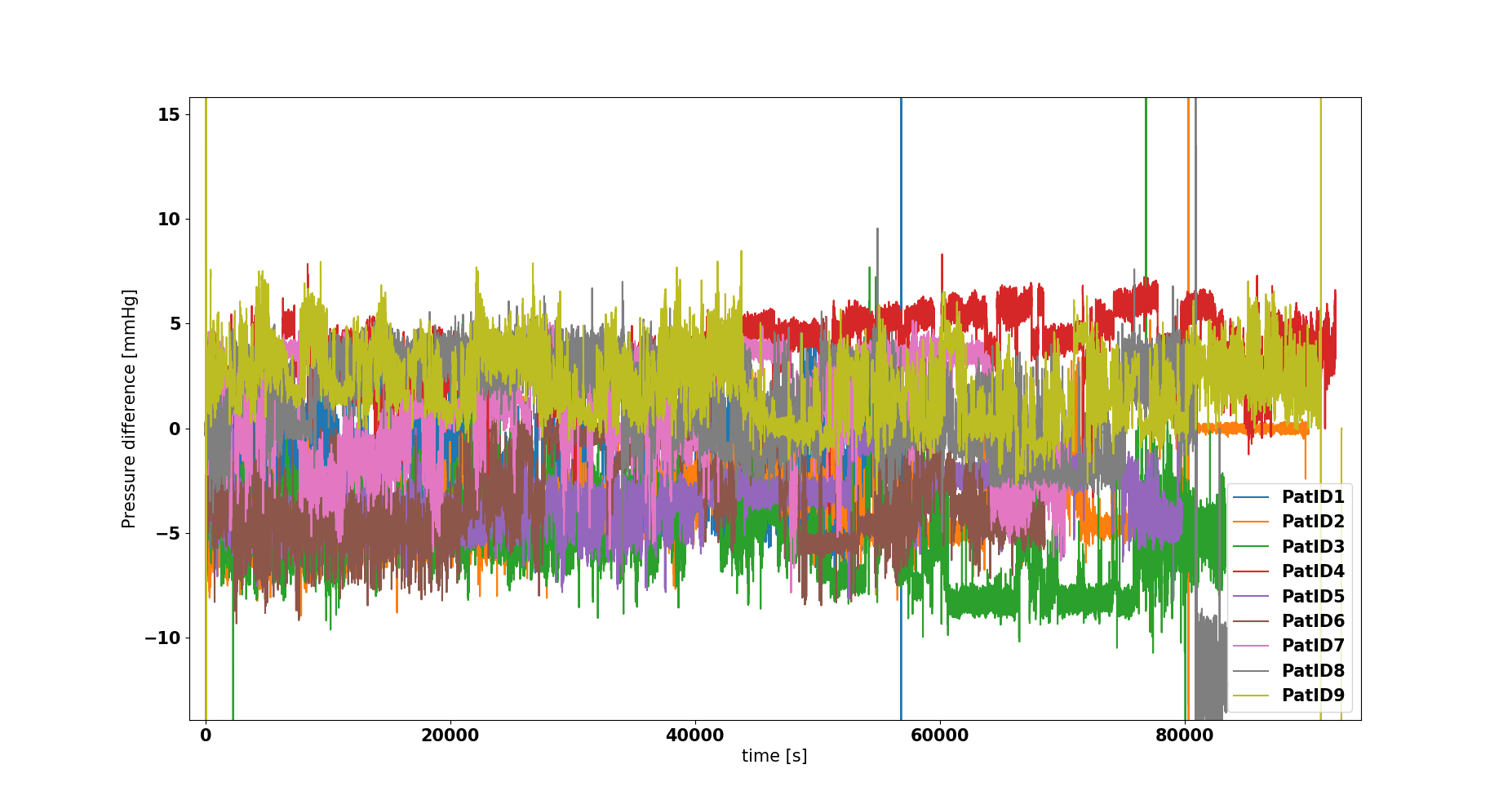
**Supplementary Figure S1:** Pressure differences for all patients over the entire period of ICP monitoring. Pressure difference curves do sudden shifts in time, possible due to head movements, shifting the relative hydrostatic pressure between the two sensors. This shift should not affect the pressure difference amplitude.

**Methods:**

**Experimental design**To obtain T1-weighted images, MRI was performed in a 3 Tesla (T) Philips Ingenia scanner (Philips Medical Systems®, Best, The Netherlands). The main acquisition parameters for the T1-weighted gradient echo volume scan were: TR/TE = shortest (typically 5.1/2.3 ms), FA = 8 degrees, and sampled 184 over-contiguous (overlapping) slices with 1 mm thickness, automatically reconstructed to 368 slices with 0.5 mm thickness.

To obtain PC-MRI of CSF flow in the cerebral aqueduct of the three patients, a region of interest (ROI) was manually defined using the software MATLAB and thereafter approved by a neuroradiologist (G.R.). In the surrounding tissue, a reference ROI was defined to compensate for potential biasing in the data set. Recorded velocities were transformed from pixels to centimeters per second by applying linear transformation on the velocity encoding and the range of pixels values. All pixel velocities in the ROI were summed for each time step and multiplied by the pixel size to achieve the volumetric flow rate. The main acquisition parameters for PC-MRI were: Repetition time (TR) = 25 ms, echo time (TE) = 15 ms, slice thickness = 4 mm, pixel size = 0.48 x 0.48 mm^2^, flip angle (FA) = 15 degrees, and velocity encoding gradient (VENC) = 10 cm/s with retrospective peripheral cardiac gating.

**Construction of patient-specific 3D-geometries**The process started by manual interaction with the colliding fronts option in VMTK to segment the CSF in the cerebral aqueduct. Then, the marching cubes algorithm was used to generate the surface of the segmented volume (Supplementary Fig. S2a), and the surface was subsequently smoothed (Supplementary Fig. S2b). The inlet and outlet of the cerebral aqueduct surface was constructed by plane clipping (Supplementary Fig. S2c) and cylindric extensions were added to the inlet and outlet to avoid boundary effects (Supplementary Fig. S2d). Finally, the geometric model was created with the mesh generator in VMTK.

**Supplementary Figure S2:** The steps in constructing the patient-specific computational geometries. (a) The surface obtained using the marching cube algorithm on the cerebrospinal fluid segmentation of the cerebral aqueduct. (b) The surface after being smoothed. (c) The surface after plane cutting. (d) The surface after cylindrical extension were added.

| PatID | G | Diag. | Age (yrs) | Duration of symptoms | Location ICP sensor | ^a^Clinical severity (NPH score) | ^b^Treatment | Clinical response |
| --- | --- | --- | --- | --- | --- | --- | --- | --- |
| 1 | F | iNPH | 66 | 3 months | Intraventricular + subdural | 9 | Conservative |  |
| 2 | F | iNPH | 70 | 4 years | Intraventricular + subdural | 11 | VP shunt | R |
| 3 | F | iNPH | 71 | 2 years | Intraventricular + subdural | 13 | VP shunt | R |
| 4 | F | iNPH | 54 | 7 months | Intraventricular + parenchymal | 14 | Conservative |  |
| 5 | F | iNPH | 74 | 2 years | Intraventricular + parenchymal | 11 | VP shunt | R |
| 6 | M | iNPH | 65 | 12 months | Intraventricular + subdural | 10 | Conservative |  |
| 7 | M | iNPH | 76 | 4 years | Intraventricular + subdural | 8 | VP shunt | R |
| 8 | M | iNPH | 80 | 1 year | Intraventricular + subdural | 9 | VP shunt | NR |
| 9 | F | iNPH | 76 | 2 years | Intraventricular + subdural | 9 | VP shunt | R |

**Supplementary Table S1**: Demographic and clinical data of the patient cohort. G: gender. Diag: diagnosis. F: female. M: male. ^a^Clinical severity (NPH score) ^b^Treatment: VP shunts were of type Codman-Hakim programmable 12 cm H2O opening pressure. R: Responder. NR: Non-responder
